# Predisposing Factors of Ischemic Colitis: Data from 14 Years of Experience in a Single Center

**DOI:** 10.1155/2017/1049810

**Published:** 2017-07-09

**Authors:** Hyun Il Seo, Kyoo-ho Choi, Koon Hee Han, Sang Jin Lee, Jong Kyu Park, Young Don Kim, Gab Jin Cheon

**Affiliations:** University of Ulsan College of Medicine, Gangneung Asan Hospital, Gangneung, Republic of Korea

## Abstract

**Background and Aims:**

While several case reports on ischemic colitis (IC) suggest the presence of predisposing causative factors, a few studies have investigated the predisposing factors in IC. This study aimed to identify the characteristics of patients with IC, particularly focusing on the predisposing factors.

**Methods:**

We conducted a single-center, retrospective analysis of 159 patients with IC. Clinical characteristics, laboratory data, endoscopic findings, and medical records were reviewed. Data were compared between groups of patients defined according to the predisposing factors. The predisposing factors are defined as temporary states or episodic events occurring within a week before the development of IC such as colonoscopy, enema, use of laxatives, heavy drinking, pancreatitis, shock, and burn.

**Results:**

Compared to the group of patients without predisposing factors of IC, the group of patients with predisposing factors was characterized by a relatively higher prevalence of male sex (56.9% versus 33.3%, *p* = 0.005), younger age (60.9 ± 15.4 versus 67.2 ± 13.4 years, *p* = 0.010), lower incidence of hypertension (43.1% versus 60.2%, *p* = 0.044), and fewer risk factors (1.24 ± 1.18 versus 1.82 ± 1.22, *p* = 0.005).

**Conclusions:**

Among men with predisposing factors, IC may develop even at a relatively younger age and in the absence of multiple risk factors, suggesting that predisposing factors may be involved in the pathogenesis of IC.

## 1. Introduction

Ischemic colitis (IC) is a medical condition that develops when blood flow to the colon is insufficient to maintain normal physiological function [1]. While IC can occur in adults of all ages [2, 3], multiple studies have confirmed that advanced age, female sex, certain comorbidities, and use of certain drugs increase the risk for developing IC [2–7]. Nevertheless, IC has also been reported among young adults without known risk factors [8–10]. In addition, many case reports proposed colonoscopy, enema, and use of some dietary supplements as predisposing factors instead of risk factors for the development of IC [8, 9, 11]. To date, the role of predisposing factors in the development of IC has not been completely understood, partly due to the lack of studies focusing specifically on such factors.

With the aim to identify the predisposing factors of IC, the present study investigated the characteristics of patients with IC treated at our hospital, which is the only tertiary-care hospital in the Mideast region (“Young dong” area) of Korea.

## 2. Methods

### 2.1. Patient Selection

The study was approved by the Institutional Review Board of Gangneung Asan Hospital (Gangneung, Korea). Using the hospital's database, we identified patients with the diagnosis code K55 (vascular disorder of the intestine) according to the International Classification of Diseases 10th version (ICD-10), who were treated between 2000 and 2014 in Gangneung Asan Hospital. Diagnosis code K55 applies in the following cases: (1) elderly patients with typical symptoms (abdominal pain with hematochezia), (2) cases of enterocolitis admitted for abdominal symptoms (such as pain, nausea/vomiting, and diarrhea) but not diagnosed as infectious enterocolitis or food poisoning based on blood and stool tests, computed tomography (CT), and/or colonoscopy, and (3) vascular (celiac, superior, or inferior mesenteric artery) occlusion observed on CT regardless of abdominal symptoms and other tests. We retrospectively reviewed the medical records of these patients to establish the likelihood of IC, based on clinical symptoms as well as endoscopic, pathologic, and radiographic findings. The following exclusion criteria were applied: (1) missing endoscopic images, (2) small bowel ischemia without colorectal involvement, (3) colorectal angiodysplasia without signs of colitis, and (4) inflammatory bowel disease. For eligible patients, the endoscopic images obtained at the time of diagnosis were reviewed independently by two gastrointestinal specialists (HI Seo and KH Han). They made the following diagnoses based on subjective judgment: definite, probable and possible IC, and not IC. In the final analysis, we only included the cases for which the diagnosis of IC was assessed as definite or probable according to both observers. Endoscopic diagnosis was based on the presence of erythema, edema, erosion or ulcer, and grey mucosa potentially indicative of mucosal necrosis. We carefully reviewed the patients' medical records to extract information regarding clinical characteristics, comorbidities, concomitant use of drugs, laboratory results, endoscopic findings, radiological findings, history of present illness on admission, and route of admission.

### 2.2. Risk Factors and Predisposing Factors

We defined risk factors as underlying or recurring conditions with a sustained effect on the patients. The following 14 conditions were included as risk factors: hypertension, diabetes mellitus, history of ischemic heart disease, stroke, deep vein thrombosis, or abdominal surgery, peripheral arterial disease, constipation, atrial fibrillation, chronic obstructive lung disease, rheumatic disease, hemodialysis, use of diuretics, and recent history of chemotherapy. The total number of risk factors in each patient was calculated.

We defined predisposing factors as temporary states or episodic events occurring within a week before the development of IC. The following events were included as predisposing factors for IC: colonoscopy, enema, episodic use of laxatives, heavy drinking, pancreatitis not related to alcohol consumption, shock (including cardiopulmonary resuscitation and sepsis), burn, and diarrhea unrelated to enema or use of laxatives. We excluded diarrhea if it occurred before hematochezia on the same day.

Patients were divided into two groups according to whether they had predisposing factors (+) or not (−). Next, we compared the clinical characteristics of the two groups.

### 2.3. Statistical Analysis

Data analysis was performed using SPSS version 21.0 (SPSS Inc., Chicago, IL). Quantitative variables were expressed as mean ± standard deviation, and categorical variables were expressed as numbers and percentages. When comparing the groups of patients defined according to the presence of predisposing factors, the chi-square test or Fisher's exact test (as appropriate) was used for categorical variables, while the *t*-test was used for continuous variables. A *p* value less than 0.05 indicated statistical significance.

## 3. Results

### 3.1. Clinical Characteristics

Among the 205 cases reviewed, 46 patients were excluded due to the following characteristics: no colonoscopy image (*n* = 12); possible or no ischemic colitis after review of the endoscopic image (*n* = 12); other diseases such as small bowel ischemia without colon involvement, angiodysplasia, inflammatory bowel disease, and pseudomembranous colitis (*n* = 18); and readmission after ischemic colitis for exam without symptoms (*n* = 4). A total of 159 patients were included in the final analysis (mean age, 65.2 ± 14.4 years; range, 24–91 years). Among the patients enrolled, 86.2% were older than 50 years, 59.1% were female, and 39.0% (62 patients) were referred to us from other hospitals or clinics. The most common symptoms on admission were rectal bleeding (86.2%) and abdominal pain (82.4%), with 71.1% of patients exhibiting both symptoms. Hypertension (54.7%), diabetes mellitus (19.5%), and constipation (17.6%) were the most common risk factors. Furthermore, 37 patients (23.3%) had a history of abdominal or pelvic surgery, including appendectomy but excluding hemorrhoidectomy. A total of 89 patients (56%) used prescription medications, among which calcium channel blockers (34.0%), aspirin (27.0%), and angiotensin-converting enzyme inhibitors or angiotensin receptor blockers (24.5%) were most common. Endoscopic findings indicated that the sigmoid colon was the most commonly affected segment (69.2%), while the ascending colon and rectum were the least affected segments (18.2% for both). Endoscopic findings varied and included erythema, edema, erosion, ulcers, and mucosal necrosis. Most patients (81.1%) had a circumferential bowel involvement, and only 10 patients (6.3%) had a colon single-stripe sign [12].

### 3.2. Laboratory Data and Treatment Outcome

The white blood cell count, hemoglobin, albumin, serum sodium, and C-reactive protein levels at admission were 12.3 ± 6.5 × 10^3^/*μ*L, 13.3 ± 2.2 g/dL, 3.8 ± 0.6 g/dL, 137.1 ± 4.7 mEq/L, and 5.0 ± 6.3 mg/dL, respectively. Antibiotics were prescribed to 124 patients (78.0%): fluoroquinolone alone (*n* = 48) or with metronidazole (*n* = 49), cephalosporin alone (*n* = 7) or with metronidazole (*n* = 18), and metronidazole alone (*n* = 2). Four patients underwent surgery for IC, one patient died of sepsis, and 4 patients were discharged in helpless condition (Table 1). The mean length of hospital stay was 11.2 ± 14.6 days. After excluding data on 14 patients who faced other major problems (not related to IC) during their hospitalization, the duration of hospital stay was only 8.5 ± 7.9 days.

### 3.3. Comparison of Clinical Characteristics between the Groups of Patients with (+) or without (−) Predisposing Factors

Compared to the predisposing factor (−) group, the predisposing factor (+) group was characterized by a relatively higher prevalence of male sex (*p* = 0.005), younger age (*p* = 0.010), and lower prevalence of hypertension (*p* = 0.044). Although the prevalence of most risk factors except hypertension did not differ significantly between the two groups, the total number of risk factors was lower in the predisposing factor (+) group (*p* = 0.005). With regard to symptoms, the prevalence of abdominal pain and hematochezia, when evaluated individually, did not differ significantly between the two groups. However, there was a significant difference between the groups when considering the incidence of abdominal pain accompanied with hematochezia (*p* = 0.019) (Table 2).

With regard to laboratory data at the time of admission, only hemoglobin levels were found to be statistically different between the two groups (*p* = 0.019), with 13.9 g/dL and 13.0 g/dL for patients in the predisposing factor (+) and (−) groups, respectively. There were no significant differences in the white blood cell count and levels of C-reactive protein, albumin, and sodium between the two groups (data not shown). Similarly, no difference was noted regarding the colonic segment involved (data not shown).

## 4. Discussion

In this retrospective single-center study, patients with IC were more likely to be elderly and female. The majority of patients with IC had comorbidities. Abdominal pain, hematochezia, and diarrhea were the most common symptoms in patients with IC. The left colon was the most commonly affected segment, while the rectum and ascending colon were the least affected segments. These findings represent general characteristics of IC and are in agreement with previously reported data [1, 2, 5–7]. However, we found that patients with predisposing factors did not exhibit these characteristics. Specifically, the patients in predisposing factor (+) group were more likely to be male, relatively younger, with fewer risk factors for IC, and less likely to exhibit typical symptoms of IC (i.e., abdominal pain with hematochezia).

In this study, we differentiated between risk factors and predisposing factors based on the duration and frequency of the conditions or events that may constitute such factors. According to the definition used in this study, repeated hemodialysis and chemotherapy were considered as risk factors, whereas a sudden increase in alcohol consumption prior to development of IC was considered a predisposing factor.

Although IC is predominant among elderly individuals, there are several reports of IC occurring in younger individuals with predisposing factors but with no risk factors [9, 10]. Taken together with these previous observations, our present findings regarding the higher prevalence of predisposing factors among relatively younger individuals with fewer risk factors suggest that the predisposing factors themselves may indeed contribute to the development of IC.

Many previous studies have referred to predisposing and/or risk factors of IC using terms such as predisposing factor/condition, risk factor, medical condition, comorbidity, underlying medical illness, or etiological factor [5, 8–11, 13, 14]. However, based on our definition, most of the conditions considered in previous studies would be classified as risk factors, not predisposing factors. Although there is no clear consensus on the use of terms: predisposing factor and risk factor, distinguishing between these two entities is clinically relevant. The majority of risk factors (such as atherosclerosis, hypertension, diabetes mellitus, rheumatic disease, or prescribed medication) represent unavoidable conditions, whereas some predisposing factors (such as alcohol consumption, enema, or colonoscopy) can be influenced. Our results suggest that most predisposing factors are related to dehydration or hypotension, which may explain the higher hemoglobin levels noted in the predisposing factor (+) group. In addition, this observation is consistent with the pathophysiology of IC, which may develop as a result of various conditions or factors reducing blood flow to the colon. As suggested by Brandt and Boley [15], colonic blood flow is very low regardless of the segment, which may explain why individuals with predisposing factors are affected even though they may be younger and have fewer risk factors. Therefore, it is likely that IC can be prevented in this population by controlling the predisposing factors; for example, performing hydration before or during enema, using less air for insufflation during colonoscopy, and using carbon dioxide rather than room air for insufflation [16].

Our results showed that typical symptoms of IC were more prevalent in the predisposing factor (−) group. In general, most patients may talk about their symptoms that occur to themselves to the doctors. However, in older patients with abdominal symptoms, the doctor may predict and request diagnosis for ischemic colitis in advance, which may explain our results.

Our study has the following limitations. First, this was a retrospective study. Although we thoroughly scrutinized the patients' medical records, it is possible that some data were overlooked. Nevertheless, scrutinizing the medical records one at a time helped us to recognize the predisposing factors in each patient. Second, although the medical records of most patients contained a pathology report, we used endoscopic findings, rather than pathology findings, to confirm the diagnosis of IC. Our reasoning was that, in practice, pathological findings are nonspecific and rarely used in the diagnosis of IC [17]. Instead, histopathological findings are mostly considered as supportive for the diagnosis of IC in the larger context of the patient's clinical presentation [1]. On the other hand, endoscopic findings are known to have high diagnostic accuracy for IC [18], which was why we based the diagnosis on endoscopic findings and excluded patients whose medical records did not contain endoscopic images. Third, this was a single-center study, and thus, our results may not be generally applicable.

## 5. Conclusion

Despite its limitations, our study demonstrated evidence that while the majority of patients with IC were elderly, had one or more risk factors, and were female, a different profile was noted for patients with predisposing factors; these patients were relatively younger, more likely to be male, and had fewer risk factors. Our findings suggest that predisposing factors, which are mostly related to dehydration, contribute to the pathogenesis of IC. Therefore, it may be possible to prevent IC by controlling the predisposing factors in this population.

## Figures and Tables

**Table 1:** Clinical characteristics of 159 patients with ischemic colitis.

Clinical characteristics	
Mean age, years	65.2 ± 14.4
≤49 years	22 (13.8)
≥50 years	137 (86.2)
Sex	
Female	94 (59.1)
Male	65 (40.9)
Symptom	
Abdominal pain	131 (82.4)
Hematochezia	137 (86.2)
Pain and bleeding	113 (71.1)
Nausea/vomiting	23 (14.5)
Diarrhea	48 (30.2)
Involved region	
Ascending colon	29 (18.2)
Transverse colon	49 (30.8)
Descending colon	84 (52.8)
Sigmoid colon	110 (69.2)
Rectum	29 (18.2)
Colonoscopy findings	
Ulcer^†^	96 (60.4)
Circumferential lesion	129 (81.1)
Colon single-stripe sign	10 (6.3)
Routes of admission	
Emergency department	97 (61)
Transfer	12 (7.5)
Outpatient clinic visits	50 (31.5)
Risk factor	
Hypertension	87 (54.7)
IHD	19 (12.0)
Diabetes mellitus	31 (19.5)
Stroke	17 (10.7)
Constipation	28 (17.6)
Abdominal surgery^‡^	37 (23.3)
Atrial fibrillation	11 (6.9)
COPD	6 (3.8)
Rheumatic disease	4 (2.5)
Hemodialysis	4 (2.5)
Diuretics	11 (6.9)
Chemotherapy	2 (1.3)
DVT	1 (0.6)
PVD	1 (0.6)
Number of risk factors	1.63 ± 1.24
Predisposing factor	51 (32.1)
Diarrhea	16 (10.1)
Alcohol consumption	11 (6.9)
Colonoscopy^§^	7 (4.4)
Enema or laxative use	11 (6.9)
Shock	4 (2.5)
Pancreatitis	1 (0.6)
Burn	1 (0.6)
Antibiotics use	124 (78)
Unfavorable outcomes^¶^	9 (5.7)

Data are given as mean ± standard deviation or total number (percentage). IHD: ischemic heart disease; COPD: chronic obstructive pulmonary disease; DVT: deep vein thrombosis; PVD: peripheral vascular disease; ^†^ulcer includes ulcer and mucosal necrosis; ^‡^abdominal surgery includes laparotomy, appendectomy, and laparoscopic surgery; ^§^colonoscopy includes colonoscopy and colonic polypectomy; ^¶^unfavorable outcomes include surgical treatment, terminal discharge, and death.

**Table 2:** Comparison between groups of patients with ischemic colitis, stratified according to the presence of predisposing factors.

Clinical characteristics	All (*N* = 159)	Predisposing factor		*p* value
		(−)	(+)	
		(*n* = 108)	(*n* = 51)	
Mean age, years	65.2 ± 14.4	67.2 ± 13.4	60.9 ± 15.4	0.010
Sex				0.005
Female	94 (59.1)	72 (66.7)	22 (43.1)	
Male	65 (40.9)	36 (33.3)	29 (56.9)	
Symptoms				
Abdominal pain	131 (82.4)	93 (86.1)	38 (74.5)	0.073
Hematochezia	137 (86.2)	95 (88.0)	42 (82.4)	0.339
Pain and bleeding	113 (71.1)	83 (76.9)	30 (58.8)	0.019
Risk factors				
Hypertension	87 (54.7)	65 (60.2)	22 (43.1)	0.044
IHD	19 (12.0)	15 (13.9)	4 (7.8)	0.273
Diabetes mellitus	31 (19.5)	24 (22.2)	7 (13.7)	0.207
Stroke	16 (10.1)	14 (13.0)	3 (5.9)	0.177
Constipation	28 (17.6)	19 (17.6)	9 (17.6)	0.993
Abdominal surgery^†^	37 (23.3)	28 (25.9)	9 (17.6)	0.249
Atrial fibrillation	11 (6.9)	10 (9.3)	1 (2.0)	0.107
COPD	6 (3.8)	3 (2.8)	3 (5.9)	0.386
Rheumatic disease	4 (2.5)	4 (3.7)	0	0.307
Hemodialysis	4 (2.5)	4 (3.7)	0	0.307
Use of diuretics	11 (6.9)	9 (8.3)	2 (3.9)	0.505
Chemotherapy	2 (1.3)	1 (0.9)	1 (2.0)	0.540
DVT	1 (0.6)	1 (0.9)	0	1.0
PVD	1 (0.6)	0	1 (2.0)	0.321
Number of risk factors	1.68 ± 1.28	1.82 ± 1.22	1.24 ± 1.18	0.005

Data are given as mean ± standard deviation or total number (percentage). *p* values are computed for the comparison between groups with and without predisposing factors; IHD: ischemic heart disease; COPD: chronic obstructive pulmonary disease; DVT: deep vein thrombosis; PVD: peripheral vascular disease; ^†^abdominal surgery includes laparotomy, appendectomy, and laparoscopic surgery.
